# Endothelial Damage in Sepsis: The Interplay of Coagulopathy, Capillary Leak, and Vasoplegia—A Physiopathological Study

**DOI:** 10.3390/clinpract15070120

**Published:** 2025-06-25

**Authors:** Gianni Turcato, Arian Zaboli, Lucia Filippi, Alessandro Cipriano, Paolo Ferretto, Michael Maggi, Fabrizio Lucente, Massimo Marchetti, Lorenzo Ghiadoni, Christian J. Wiedermann

**Affiliations:** 1 Intermediate Care Unit, Department of Internal Medicine, Hospital Alto Vicentino (AULSS-7), 36014 Santorso, Italy; lucia.filippi@aulss7.veneto.it (L.F.); paolo.ferretto@aulss7.veneto.it (P.F.); michael.maggi@aulss7.veneto.it (M.M.); fabrizio.lucente@aulss7.veneto.it (F.L.); massimo.marchetti@aulss7.veneto.it (M.M.); 2 Department Health Science, UniCamillus-Saint Camillus International University of Health Sciences, 00131 Rome, Italy; 3 Innovation, Research and Teaching Service (SABES-ASDAA), Teaching Hospital of the Paracelsus Medical Private University (PMU), 39100 Bolzano, Italy; zaboliarian@gmail.com; 4 Emergency Department, Nuovo Santa Chiara Hospital, Pisa University Hospital, 56126 Pisa, Italy; alessandrocipriano@gmail.com; 5 Department of Clinical and Experimental Medicine, University of Pisa, 56127 Pisa, Italy; lorenzo.ghiadoni@unipi.it; 6 Institute of General Medicine and Public Health, Claudiana, 39100 Bolzano, Italy; christian.wiedermann@am-mg.claudiana.bz.it

**Keywords:** sepsis, endothelial dysfunction, capillary permeability, sepsis-induced coagulopathy, albumins

## Abstract

**Background:** Sepsis remains a leading cause of mortality worldwide, and understanding endothelial damage is crucial for improving patient outcomes. Endothelial dysfunction in sepsis contributes to coagulopathy, increased capillary permeability, and vasoplegia, but the interplay between these processes remains underexplored. The study aims to evaluate the clinical relationship between those factors due to sepsis-induced endothelial damage. **Methods:** A prospective single-center study on 75 community-acquired septic patients admitted to an Intermediate Care Unit. The Sepsis-Induced Coagulopathy (SIC) score, serum albumin (as a surrogate for capillary leak), and Total Peripheral Resistance Index (TPRI) (as a surrogate for vasoplegia) were assessed. Structural Equation Modeling (SEM) explored the relationship between variables, hypothesizing a common latent factor (endothelial damage). Principal Component Analysis assessed the shared variance among variables. **Results:** The mean SIC score was 3.4 (SD 1.3), with 44% of patients affected. TPRI and albumin had mean values of 1954 (SD 738) and 2.58 (SD 0.59), respectively, both negatively correlated with SIC: TPRI −0.263 (*p* = 0.023) and albumin −0.454 (*p* < 0.001). SEM showed SIC, albumin, and TPRI are associated with a latent factor (endothelial damage), explaining 68% of the variance (CFI = 1.000, RMSEA = 0.000). Albumin was inversely correlated (*p* = 0.004), and TPRI was significantly associated (*p* = 0.003). **Conclusions:** This pilot study suggests that coagulopathy, increased vascular permeability, and vasoplegia may be clinically interrelated manifestations of endothelial injury in sepsis. These findings support the feasibility of modeling a unified pathophysiological construct using accessible bedside data, potentially guiding future individualized approaches in sepsis management.

## 1. Introduction

Sepsis remains a complex clinical syndrome and one of the main causes of morbidity and mortality worldwide [1,2]. Despite significant advances in early identification and therapeutic strategies, the ability to tailor interventions to specific underlying mechanisms is still limited [3,4]. This limitation stems from the disconnect between well-characterized pathophysiological models and the clinical tools currently available, which are often generic and non-individualized [4].

A central and well-established component of sepsis pathophysiology is endothelial dysfunction, which plays a pivotal role in promoting the three hallmark alterations of advanced sepsis: coagulopathy, increased capillary permeability, and vasoplegia [5,6,7]. Each of these alterations can be observed clinically and contributes independently to organ dysfunction and prognosis. However, despite the clear theoretical links, they are often studied and managed as separate entities.

While direct assessment of endothelial integrity is not yet feasible in clinical practice, particularly in acute settings, several readily measurable parameters have been shown to indirectly reflect the severity of these dysfunctions. The Sepsis-Induced Coagulopathy (SIC) score is an early and sensitive marker of pro-thrombotic imbalance and has been associated with mortality in septic patients [8]. Serum albumin, long recognized as a nutritional marker, is increasingly considered a surrogate of endothelial permeability, as its reduction reflects vascular leakage in sepsis [9,10]. Finally, Total Peripheral Resistance Index (TPRI), assessable through non-invasive hemodynamic monitoring, offers insight into vasomotor dysregulation, a key component of septic vasoplegia [11].

Although these three dysfunctions are typically approached as separate clinical issues, their shared pathophysiological origin in endothelial injury suggests the possibility of an integrated model. The present study aims to explore this hypothesis by assessing whether coagulopathy (SIC score), capillary leak (serum albumin), and vasoplegia (TPRI) can be understood as interrelated expressions of a single latent construct: endothelial damage.

Our objective is to evaluate whether this theoretical construct can statistically account for the variability observed in these three clinical parameters. This approach may offer a preliminary framework for translating complex pathophysiological mechanisms into practical bedside models, ultimately supporting a more mechanistic and individualized evaluation of sepsis severity.

## 2. Objectives

The aim of this pilot study is to assess the feasibility of developing an integrated model that links three clinically observable dysfunctions (coagulative alterations, increased capillary permeability, and vasoplegia) to a single latent factor (endothelial damage), which is currently not quantifiable in clinical practice. This approach is intended to lay the groundwork for future clinical investigations that, for the first time, will utilize measurable clinical variables to infer underlying pathophysiological mechanisms, thereby opening up new diagnostic and therapeutic perspectives.

## 3. Methods

### 3.1. Study Design

A prospective observational pilot study was conducted between 1 September 2024 and 31 December 2024, at the Intermediate Medical Care Unit (IMCU) of the Alto-Vicentino Hospital in Santorso.

### 3.2. Study Hypotheses

This pilot study was designed to explore the following hypotheses:

Primary Hypothesis: The underlying assumption is that sepsis follows a pathophysiological pattern in which a common latent factor, currently difficult to quantify clinically (endothelial damage) triggers the sepsis-induced alterations of the coagulative system, vascular permeability, and vasomotor function. These three conditions are more definable from a clinical standpoint, with surrogates that have been extensively studied in recent years and are obtainable in daily clinical practice.

Secondary Hypothesis: Once initiated by the common factor, the three conditions may influence each other and worsen.

Tertiary Hypothesis: These conditions may present at varying levels in septic patients and display clusters.

### 3.3. Patients

The study included all patients admitted from the Emergency Department (ED) to the IMCU for community-acquired sepsis. Sepsis was defined following the most recent Surviving Sepsis Campaign guidelines, characterized as an infection that is either confirmed or suspected and is associated with organ damage, the latter determined by the Sequential Organ Failure Assessment (SOFA) score [4]. The definition of community-acquired was limited to those not recently hospitalized (within the last four weeks), and those who had not undergone major surgery or experienced significant trauma, including recent fractures, during the same period.

Patients were excluded from the study if they were under 18 years old, pregnant, or if they had been in the ED for more than six hours before being admitted to the IMCU. This time-based criterion was adopted to reduce the impact of prolonged pre-IMCU stabilization, which could include significant fluid administration, vasopressor use, or other therapeutic interventions likely to influence serum albumin concentrations and non-invasive hemodynamic parameters, thus confounding their interpretability as early indicators of endothelial dysfunction. Additional exclusion criteria were the administration of more than 1000 mL of crystalloids within three hours before admission to the IMCU, any prior administration of colloids, acute bleeding (confirmed or suspected), concurrent major trauma or acute post-traumatic fractures, and patients deemed by IMCU physicians to have a low probability of survival within the next 24 h. Those with terminal oncological illnesses expected to survive less than three months, as assessed through clinical judgment incorporating patient history, physical examination, functional status, and current symptoms, were also excluded. Furthermore, any patient transferred to the IMCU from a department other than the ED was not considered for inclusion in the study. These exclusion criteria were selected to minimize the confounding effects of early fluid resuscitation or prolonged pre-IMCU stabilization, which may significantly alter serum albumin concentrations and non-invasive hemodynamic measurements such as TPRI. The aim was to preserve a more homogeneous baseline for the pathophysiological modeling.

Patient enrolment was performed consecutively, including all eligible cases 7 days a week. No patients were excluded due to weekend or night-time limitations, as dedicated clinical staff ensured consistent coverage during the study period.

### 3.4. Study Protocol and Variables

Upon enrollment, patients underwent simultaneous comprehensive blood testing including complete blood count, coagulation profile (PT-INR, aPTT, fibrinogen, and D-dimer), renal function and electrolytes, liver function tests, inflammatory markers (C reactive protein [CRP], procalcitonin [PCT]), and serum albumin levels, along with arterial blood gas analysis and non-invasive hemodynamic assessment using the NI-MEDICAL NICaS^®^ system.

Serum albumin has been extensively studied in recent years as a potential indicator of capillary leakage [10,12,13]. Its reduction, associated with negative short-to-medium term outcomes, has been linked to impaired normal exchange between vessels and interstitial spaces [10,12,13]. In this pilot study, serum albumin levels were considered as a clinical surrogate for altered capillary permeability. Serum albumin was used as a clinical surrogate for capillary leak, based on its established association with endothelial permeability in sepsis. While we acknowledge that hypoalbuminemia can result from multiple causes, we applied strict exclusion criteria to reduce confounders (e.g., major fluid resuscitation, recent surgery or trauma, terminal illness) and to enhance the interpretability of albumin as an indicator of vascular leakage in this setting.

Prothrombin time (PT), platelet (PTL) count, and SOFA scores were used to construct the SIC score, which served as a clinical surrogate for the presence of sepsis-induced coagulation alterations in the study.

The SIC score was selected as the primary marker of coagulation dysfunction in this study because it is specifically designed for early identification of coagulopathy in sepsis and endorsed by the ISTH. Unlike the full ISTH-DIC score, which is used to diagnose overt disseminated intravascular coagulation and includes additional laboratory markers, the SIC score was specifically developed for septic patients and relies only on simple and easy-to-obtain laboratory and clinical parameters. These parameters are rapidly and routinely available at admission, even in non-ICU settings, making SIC more suitable for early-phase assessment in our study population.

This practical advantage made SIC particularly suitable for the clinical and organizational context of this pilot study. No missing data imputation was necessary, as all variables required for SIC score calculation were available for every patient.

Furthermore, peripheral vascular resistances were used as a clinical surrogate for the condition of altered vasomotor–capillary function caused by sepsis using the TPRI provided by the non-invasive NI-MEDICAL NICaS^®^ (NI Medical Ltd., Ra’anana, Israel) system version 3.63.15. While TPRI was considered in this study as an indirect marker of vasoplegia, we acknowledge that systemic vascular resistance may also be affected by other factors unrelated to endothelial dysfunction, including baseline cardiovascular status, medications, and autonomic tone. However, the strict exclusion criteria and the timing of measurement at IMCU admission aimed to reduce these potential confounders and enhance the interpretability of TPRI as a pathophysiological indicator in this specific setting. The NICaS^®^ is a hemodynamic monitoring device that utilizes bioelectrical impedance analysis to deliver accurate, real-time assessments of hemodynamic parameters and fluid status of the patient. Measurements were taken upon the patient’s arrival at the IMCU, with the subject in a supine position and sensors applied to the volar surfaces of both wrists. Recorded parameters included Systolic Index (SI), Cardiac Index (CI), TPRI, Total Body Water (TBW), and Cardiac Power Index (CPI). These measurements have been previously validated in studies comparing non-invasive NICaS^®^ readings with invasive thermodilution data, demonstrating the reliability of the device in hemodynamic function assessment. Moreover, if the obtained trace was inadequate, the test was repeated for up to 10 min; if a satisfactory measurement could not be obtained within this interval, the patient was excluded from the study.

### 3.5. Ethical Consideration

The study was approved by the local ethics committee (Clinical Trial Ethics Committee ULSS 8, Berica-Vicenza, Italy; approval number: n.19814/23) and was conducted in accordance with the ethical principles for medical research involving human subjects as defined by the Declaration of Helsinki.

## 4. Statistical Analysis

No formal a priori sample-size calculation was conducted, as this was an exploratory pilot study based on consecutive patient enrollment. The objective was to assess the conceptual and statistical feasibility of a latent model of endothelial dysfunction using accessible clinical parameters.

Categorical variables were described as percentages and the number of events relative to the total, while continuous variables were reported as means and standard deviations or medians and interquartile ranges, depending on their distribution. Univariate analyses were conducted using Fisher’s exact test, Chi-square, Student’s *t*-test, Kruskal–Wallis, and Mann–Whitney tests.

Hypothesis 1 (The underlying assumption is that sepsis follows a pathophysiological pattern driven by a common latent factor) was explored using Structural Equation Modeling (SEM). SEM is a multivariate technique that models causal relationships between observed and latent variables, integrating both direct measurements and theoretical assumptions about the data structure. In the SEM models, the latent variable (endothelial alteration) was posited as the common driver of variations in the three clinical variables (serum albumin; TPRI; and SIC score). For model construction, the variance of the latent variable was fixed at 1 to ensure model identification, as is standard practice in structural equation modeling. Relationships between the latent variable and the clinical variables were then estimated as regression coefficients, with residuals of the clinical variables modeled to capture unexplained variance. Model fit was assessed using multiple indices: Root Mean Square Error of Approximation (RMSEA), Standardized Root Mean Square Residual (SRMR), Comparative Fit Index (CFI), the Tucker–Lewis Index (TLI), and Coefficient of Determination (CD). Values of RMSEA and SRMR < 0.05 and CFI/TLI > 0.95 were considered indicative of excellent model fit. Considering SEM’s sensitivity to sample size and distributional assumptions, diagnostic tests were conducted to assess the distributional characteristics of the observed variables. Deviations from normality were evaluated in light of the model complexity and sample size, and were deemed acceptable for SEM in small-to-moderate clinical samples.

Given the sensitivity of SEM to sample size and distributional assumptions, particular attention was paid to ensuring model adequacy. According to established guidelines, a minimum sample-to-parameter ratio of 10:1 is recommended to ensure reliable estimates [14]. Our model included 7 freely estimated parameters (3 path coefficients, 3 residual variances, and 1 intercept), requiring a minimum of 70 subjects. With 75 patients, our analysis satisfied this criterion. Additionally, the observed variables were evaluated for approximate normality using skewness and kurtosis. While some moderate deviation was observed, particularly for the SIC score, SEM is known to tolerate such non-normality in small-to-moderate samples.

Hypothesis 2 (the interdependence of the three clinical parameters) was initially explored by assessing the univariate correlations among the clinical variables (serum albumin, TPRI, and SIC score), reported using Pearson’s correlation coefficients. Principal Component Analysis (PCA) was employed to analyze potential interconnections among the clinical variables. PCA is a statistical technique used to reduce the dimensionality of a dataset of correlated variables into a smaller number of uncorrelated principal components, which explain the total variance and reveal underlying structures or latent patterns while preserving as much information as possible.

Hypothesis 3 (the presence of severity clusters) was evaluated using a dendrogram-based hierarchical cluster analysis to determine if the interacting clinical variables could characterize specific patient groups, suggesting a structured relationship among the variables. Any identified clusters were then compared with the short-to-medium-term outcomes of the patients. Analysis of variance was conducted across the possible clusters, and Bartlett’s test was applied.

All analyses were performed using STATA 16.1, and a *p*-value of less than 0.05 was considered statistically significant.

## 5. Results

A total of 89 patients admitted to the IMCU with community-acquired sepsis were assessed for study eligibility. Of these, 14 were excluded based on predefined criteria, and 75 septic patients were enrolled in the pilot study (Supplementary File S1). The characteristics of the patients are listed in Table 1. The mean SIC score was 3.4 (SD 1.3), with 43.9% (33/75) of patients exhibiting coagulopathy at the time of enrollment. The mean TPRI was 1954 (SD 738), with 42.7% (32/75) of patients presenting a TPRI below the normal range. The mean serum albumin level at admission was 2.57 g/dL (SD 0.58), with 48% of patients (36/75) having an albumin level < 2.5 g/dL at admission.

Hypothesis 1: The results of the SEM analysis demonstrated an excellent fit between the hypothesized model and the observed data. The latent factor in the model significantly influenced all three clinical variables recorded at admission. Prior to model estimation, the distributional properties of the observed variables were assessed through Shapiro–Wilk tests, skewness, and kurtosis, as recommended for SEM applications. The results of these diagnostics are provided in Supplementary File S2. Global fit indices confirmed the model’s robustness and accuracy in reproducing the observed relationships: RMSEA = 0.000 (*p* < 0.01), SRMR = 0.000 (*p* < 0.01), CFI = 1.000, and TLI = 1.000, all indicating an excellent goodness of fit.

**Figure 1:**
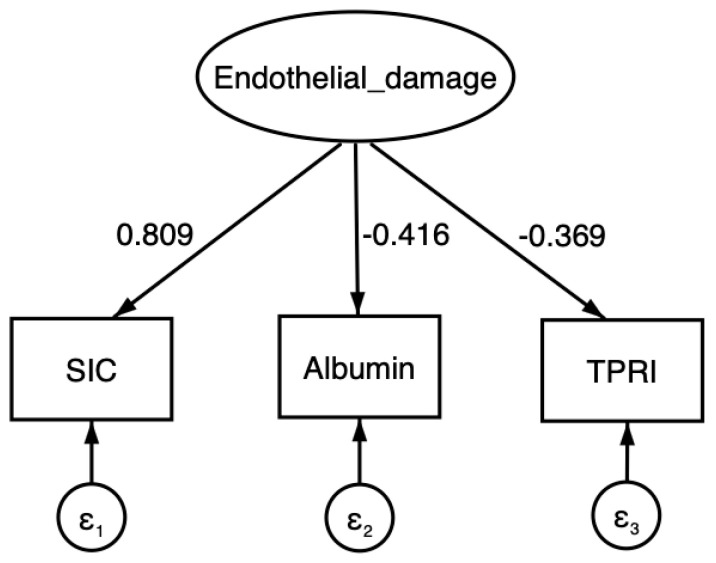
Structural equation model illustrating the relationship between endothelial damage and clinical parameters. Endothelial damage is positively associated with the SIC score and negatively associated with serum albumin levels and TPRI. The model includes error terms (ε_1_, ε_2_, ε_3_) corresponding to each observed variable.

The hypothesized model appears to explain 67.9% of the total variance in the observed variables (coefficient of determination = 0.679). The relationships identified through SEM suggest that an increase of one hypothetical unit in the latent factor is associated with an increase of 0.809 units in the SIC score (*p* < 0.001), a decrease of 0.416 units in serum albumin (*p* < 0.001), and a decrease of 0.369 units in TPRI (*p* < 0.001) (Figure 1, Table 2).

Hypothesis 2: The correlations among the indicator variables are listed in Table 3.

**Figure 2:**
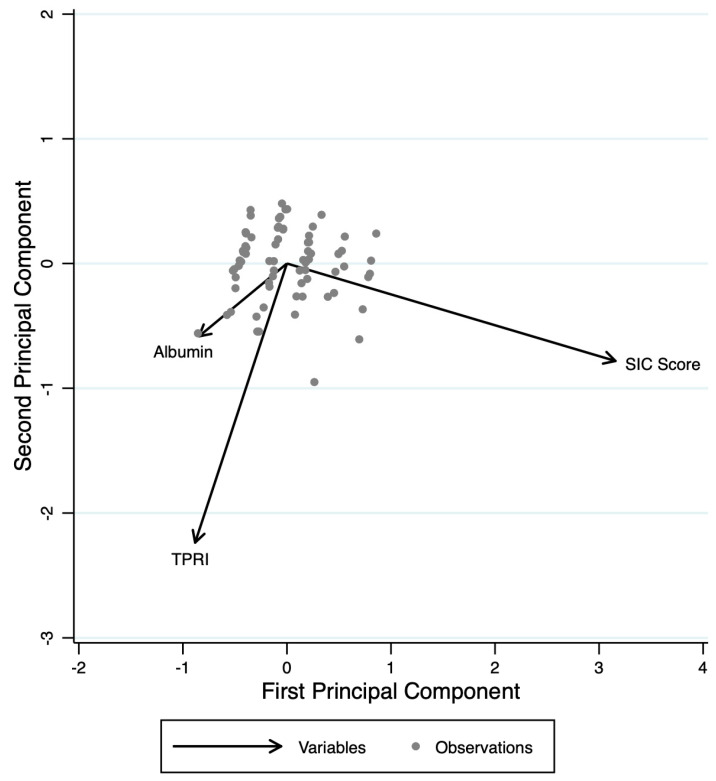
PCA biplot illustrating the relationships between SIC Score, Albumin, and TPRI. The arrows represent the direction and magnitude of each variable’s contribution to the first and second principal components. SIC Score shows a strong positive correlation with the first principal component, while Albumin and TPRI are negatively associated. The grey dots represent individual observations within the dataset.

PCA identified three main components that together explain 100% of the total variance of albumin, TPRI, and SIC score (Figure 2). The first component (Comp1, eigenvalue = 1.768) accounts for 58.95% of the variance and is characterized by a strong positive contribution from albumin (loading = 0.606) and TPRI (loading = 0.5338), while the SIC score contributes negatively (loading = −0.5898). Comp1 appears to represent a dimension of capillary and vasoplegic dysfunction. The second component (Comp2, eigenvalue = 0.697) explains an additional 23.24% of the variance and is primarily driven by a strong positive contribution from TPRI (loading = 0.8366) and SIC score (loading = 0.4725), while albumin contributes negatively (loading = −0.2772). Comp2 mainly reflects a hemodynamic axis, primarily influenced by TPRI. The third component (Comp3, eigenvalue = 0.534) accounts for the remaining 17.81% of the variance and is dominated by albumin (loading = 0.7457) and SIC score (loading = 0.6549), with a minimal and negative contribution from TPRI (loading = −0.1228). Comp3 suggests a coagulation–capillary dimension. The first two components together explain 82.19% of the total variance, indicating that the variability of these three variables is largely captured by these two main axes. This finding highlights the fact that while the three dimensions are interrelated, they represent distinct aspects of the hypothesized model.

Hypothesis 3: Cluster analysis identified three distinct patient groups (Table 4). Cluster 1 (n = 39) appears to represent an intermediate pathophysiological state, characterized by moderate SIC scores (3.7 ± 1.2), low albumin levels (2.4 ± 0.5 g/dL), and intermediate TPRI values (1397 ± 318).

Cluster 2 (n = 25) includes patients with lower SIC scores (3.0 ± 1.2), higher albumin levels (2.6 ± 0.6 g/dL) compared to Cluster 1, and significantly higher TPRI values (2273 ± 176, *p* < 0.001), indicating a distinct hemodynamic profile. Notably, no deaths were observed in this cluster at 30 days.

Cluster 3 (n = 11) consists of patients with the highest TPRI values (3264 ± 466, *p* < 0.001) and elevated albumin levels (3.0 ± 0.6 g/dL, *p* = 0.004), suggesting a different pathophysiological profile. The 30-day mortality was highest in Cluster 1 (17.9%), followed by Cluster 3 (9.1%), while no deaths occurred in Cluster 2 (*p* = 0.059).

ANOVA confirmed significant differences among the clusters (*p* < 0.001), with homogeneous variances according to Bartlett’s test. The 30-day mortality was 17.9% in Cluster 1, 9.1% in Cluster 3, and 0% in Cluster 2. While overall comparison among the three clusters showed a trend toward significance (*p* = 0.059), post hoc analysis revealed a significant difference between Cluster 1 and Cluster 2 (*p* = 0.037).

## 6. Discussion

This pilot study was designed to explore the clinical hypothesis that a common latent factor (endothelial dysfunction/injury) underlies the three key pathophysiological dysfunctions characteristic of sepsis: coagulopathy, capillary permeability alterations, and vasoplegia. Although speculative pathophysiological models clearly define how endothelial cell impairment gives rise to these dysfunctions, clinically deducible and quantifiable integrated models based on accessible parameters remain scarce and are not widely validated [9,15]. This gap limits the development of clinical tools that could guide the assessment and treatment of septic patients, thereby contributing to the persistently high mortality rates associated with this condition. If confirmed, the ability to use clinical parameters, considered surrogates of the major pathophysiological dysfunctions of sepsis and readily obtainable during the initial patient evaluation, to model and “quantify” the underlying cellular alterations could offer new perspectives for targeted therapeutic interventions.

Advanced SEM analysis used for this aim demonstrated the plausibility of a model in which a common latent factor, representing endothelial injury, integrates sepsis-induced coagulation dysfunction, capillary permeability alterations, and vasomotor dysregulation. Preliminary results showed excellent model fit, confirming that these three dysfunctions share a significant relationship with the latent factor. Furthermore, SEM allowed for a quantitative estimation of the influence of endothelial injury on each of these components, supporting the concept of a unifying pathophysiological mechanism.

There is substantial evidence regarding the impact of clinical parameters on sepsis severity. The ISTH criteria for identifying SIC are well known for their ability to predict a high risk of adverse short- to medium-term outcomes [8,16]. Although these criteria were not explicitly validated for actual thrombo–hemorrhagic events or direct coagulation alterations, they have been used to assess 30-day mortality risk and are recognized for identifying a coagulopathy state triggered by endothelial dysfunction secondary to cytokine dysregulation [8,16].

A recent study by Chen et al. reported that the 28-day mortality rate was significantly higher in patients with SIC (38.6%) compared to those without SIC (20.0%, *p* < 0.001) [17]. According to Li et al., persistently elevated SIC scores (defined as SIC ≥4)m calculated repeatedly during the course of sepsis, were associated with a significantly higher 28-day mortality risk [18]. Moreover, the combined assessment of SIC and SOFA scores provided better predictive accuracy compared to either score used in isolation [19]. This synergy may be explained by the complementary nature of the two scores: while SIC captures early endothelial-driven coagulopathy, SOFA reflects the extent of systemic organ dysfunction. Their integration provides a broader and more dynamic assessment of disease severity.

Markers of endothelial injury have been widely associated with the presence of SIC [16,19]. Soluble thrombomodulin (sTM), which reflects endothelial glycocalyx degradation, is linked to the loss of the endothelium’s antithrombotic function [20]. Meanwhile, plasminogen activator inhibitor-1 (PAI-1), released by endothelial cells, inhibits fibrinolysis, thereby promoting microthrombus formation in small vessels [21].

Another key molecule released following endothelial glycocalyx disruption is angiopoietin-2 (Ang-2) [20,21]. Ang-2 promotes a procoagulant state by stimulating the release of molecules such as tissue factor and enhancing leukocyte adhesion [21,22]. At the same time, Ang-2 counteracts the protective effects of angiopoietin-1 (Ang-1) on the Tie-2 receptor, leading to increased vascular permeability and facilitating the extravasation of fluids and plasma proteins, including albumin, into the interstitial space [21,22]. The intricate connections between endothelial injury, coagulopathy, and increased capillary permeability, well-defined in pathophysiological models, may underlie the clinical correlation between elevated SIC scores and reduced serum albumin levels. McMullan et al. demonstrated that elevated levels of angiopoietin-2 (Ang-2) and syndecan-1 were associated with clinical manifestations typical of increased capillary permeability, including tissue edema, refractory hypotension, and reduced serum albumin, which is now recognized as an indirect marker of vascular barrier integrity loss [23].

Previously, Fernández-Sarmiento et al. reported a correlation between low albumin levels and elevated Ang-2 levels, suggesting that hypoalbuminemia reflects capillary leak syndrome mediated by glycocalyx degradation [24]. The unregulated translocation of protein-rich fluid into the interstitium leads to intravascular hypovolemia and tissue hypoperfusion [24].

Our findings may also be considered within the broader framework of macro-/microcirculatory coherence, a concept that emphasizes the dissociation between systemic hemodynamic parameters and microvascular/endothelial function in sepsis. Several studies have shown that even when macro-hemodynamic targets are achieved, microcirculatory perfusion remains impaired, underscoring the centrality of endothelial health in sepsis outcomes [6,7,22,23].

Although our study did not include direct measurement of endothelial biomarkers, the associations observed between SIC, albumin, and vascular resistance are consistent with known endothelial mechanisms, such as glycocalyx degradation, inflammatory-mediated permeability shifts, and dysregulation of vascular tone. Nevertheless, we recognize the value of incorporating more specific biomarkers of endothelial injury which have shown strong associations with microvascular permeability and coagulopathy in sepsis. These molecules provide more direct insight into glycocalyx degradation and endothelial activation, and their future inclusion could help refine the biological specificity of the model and validate its underlying assumptions. While this pilot phase was designed around accessible clinical parameters to assess feasibility, we agree that the integration of endothelial biomarkers represents a key next step in strengthening the translational potential of this framework.

In this context, the potential clinical relevance of our findings may extend to current therapeutic approaches that aim to protect or modulate endothelial function. For instance, the stabilizing effects of albumin on vascular permeability, as well as the endothelial benefits observed in plasma-based resuscitation or low-dose corticosteroid regimens, have been suggested in recent studies. While these were not evaluated here, our results may help inform future clinical investigations by providing a conceptual link between measurable bedside parameters and underlying endothelial dynamics.

Burgdorff et al. highlighted the fact that this reduction in effective circulating volume compromises tissue perfusion and triggers an ineffective compensatory response, characterized by persistent vasodilation and refractory hypotension [19].

Additionally, Saravi et al. demonstrated that endothelial dysfunction exacerbates this process by stimulating excessive nitric oxide (NO) production through the activation of inducible nitric oxide synthase (iNOS), driven by pro-inflammatory cytokines such as TNF-α and IL-1β [9]. This further reduces vascular tone and decreases sensitivity to vasoconstrictors, perpetuating the cycle of vascular instability in sepsis.

The combination of capillary leak and vasoplegia creates a vicious cycle of hemodynamic instability, further worsening the prognosis of septic patients [15,23]. As demonstrated by Wexler et al., in their cohort of 95 patients with severe sepsis, a lower brachial artery hyperemic velocity, measured non-invasively, was associated with increased in-hospital mortality. This finding underscores the role of hyperemic velocity as an indicator of microvascular dysfunction [25].

Similarly, Nelson et al. conducted a study involving 17 patients with sepsis or septic shock and 16 healthy controls, highlighting the fact that post-cuff occlusion flow-mediated dilation serves as a reliable marker of NO bioavailability and vascular function [26]. When combined with passive leg raising, this measure provides further insight into the vascular responsiveness and functionality in septic patients [26].

In recent years, non-invasive hemodynamic assessment has been widely explored as a strategy to improve sepsis management, particularly for guiding fluid resuscitation [27,28]. While no definitive recommendations exist, the availability of non-invasive monitoring tools represents a significant advancement in septic patient care.

For the first time, this pilot study hypothesized that sepsis-induced vasoplegic dysfunction, inferred from reduced systemic vascular resistance, could be incorporated into a multidimensional pathophysiological clinical evaluation and linked to other key aspects of sepsis pathophysiology.

Valeanu et al. reported that patients with low SVR values had a significantly higher risk of mortality, with a 20% increase in 30-day mortality rates compared to patients with normal SVR, highlighting the prognostic value of hemodynamic monitoring [28]. Similarly, Virág et al. found that in patients with septic shock, an SVR < 800 dynes·s·cm^−5^ was associated with an in-hospital mortality rate exceeding 50% [29]. This pilot study suggests that it may be both possible and necessary to integrate these findings by incorporating easily accessible clinical parameters into unifying pathophysiological models for sepsis.

By utilizing markers such as SIC score, serum albumin levels, and peripheral vascular resistance, all of which are obtainable early in the course of treatment, it is possible to derive detailed insights into the severity and progression of the disease process.

These multidimensional models could provide clinicians with a rapid and comprehensive assessment, facilitating a personalized and targeted approach, with the potential to improve patient outcomes.

In our view, this work represents the first study attempting to integrate all these parameters into a more comprehensive perspective on the septic patient and the complications associated with early endothelial dysfunction/injury. The implementation of these strategies requires further studies to validate their clinical utility and integrate them into the routine management of sepsis. However, the preliminary results of our study pave the way for an innovative approach to the evaluation and management of septic patients.

## 7. Limitations

This study has some limitations. First, its single-center design makes it susceptible to biases typically associated with this study type. However, the rigorous patient selection process and the unidimensional approach aimed to minimize these biases, while still allowing the study to achieve its predefined objectives. The intent of this study is not to provide definitive evidence, but rather to explore the feasibility of integrating pathophysiological models into clinical practice to derive valuable insights into the status and severity of cellular alterations in sepsis.

Second, the clinical surrogates for pathophysiological dysfunctions were selected a priori. However, in recent years, strong evidence has reinforced the role of the SIC score, serum albumin levels, and the TPRI in assessing sepsis severity. Despite pivotal studies demonstrating dysfunction in endothelium-mediated vasomotor tone in septic patients [24,25], non-invasive tests such as flow-mediated dilation, commonly used in cardiovascular populations, were not feasible in our setting [29,30]. Because patients were clinically unstable, these procedures were not ethically or logistically appropriate in the acute phase of care.

A third limitation is the small sample size. However, such a comprehensive and simultaneously complex approach required a highly selective sampling process to prevent excessive heterogeneity in the population, which could have led to interpretative errors. This strength inevitably results in a reduced number of enrollable patients. Nevertheless, the study’s aim is not to provide definitive evidence, but rather to explore the feasibility of integrating pathophysiological models into clinical practice to derive useful insights into the status and severity of cellular alterations occurring in sepsis. This limitation aligns with the study’s objectives, which do not seek to establish conclusive evidence, but rather to generate hypotheses for future dedicated prospective studies.

In addition, the application of SEM in a relatively small sample raises methodological considerations. Although SEM requires careful attention to sample size and variable distribution, the model used was intentionally simple (three observed indicators, one latent factor), meeting widely accepted criteria for model identification (subject-to-parameter ratio ≥10:1). Furthermore, the normality of the observed variables was formally assessed using Shapiro–Wilk tests, skewness, and kurtosis. Despite a moderate deviation in the SIC score, all diagnostics supported the appropriateness of the SEM approach in this exploratory context. The excellent fit indices further reinforced the internal consistency of the model.

Fourth, the function of the endothelium in critically ill patients can be influenced by multiple confounding factors beyond sepsis itself. Pre-existing conditions such as chronic inflammation, cardiovascular diseases, diabetes, and other comorbidities may independently affect endothelial function, potentially impacting the interpretation of the selected markers. While efforts were made to control for major confounding variables through patient selection and statistical adjustments, residual confounding cannot be entirely excluded.

Finally, we recognize that the exclusion of patients who received >1 L of crystalloids or who spent more than 6 h in the ED may have led to underrepresentation of the most critically ill septic patients. While this choice allowed for a more physiologically coherent and less-confounded study cohort, it may limit the external validity of the findings and should be considered in the interpretation of our results.

We acknowledge that serum albumin is a non-specific marker and may be influenced by chronic diseases, protein loss, or malnutrition. However, the exclusion of patients with conditions likely to impact albumin levels independently of acute sepsis, along with literature supporting its role as a marker of capillary leak, supports its use in this exploratory model. Given the exploratory design and the focused pathophysiological hypothesis, additional stratified analyses were not performed at this stage but will be considered in future confirmatory research.

Moreover, only 30-day mortality was assessed as the principal outcome measure. Other clinical endpoints such as vasopressor-free days, ICU length of stay, or need for renal replacement therapy were not consistently available across the cohort, due to the IMCU setting and the pilot nature of the study. While this limits the assessment of patient trajectories, 30-day mortality was chosen as a robust and objective outcome for early-phase evaluation.

## 8. Future Directions and Clinical Translation

This study represents an initial attempt to explore whether key clinical parameters, such as the SIC score, serum albumin, and peripheral resistance, can be statistically integrated to reflect a shared pathophysiological process consistent with endothelial injury in sepsis.

The proposed model is to be considered preliminary and hypothesis-generating. Its validity must be tested in future studies with larger, more heterogeneous populations and possibly with the inclusion of additional variables such as direct endothelial biomarkers or time-dependent measures. In particular, the incorporation of biomarkers could enhance the model’s biological specificity and mechanistic accuracy, strengthening its translational value in future studies.

Further investigation is also needed to assess the model’s reproducibility, clinical stability across different care settings, and potential association with outcome trajectories. If confirmed, this framework could support a more structured and physiology-informed stratification of septic patients in the early phase of care.

## 9. Conclusions

This pilot study suggests that clinically accessible parameters could support the development of unifying clinical models that integrate the major pathophysiological dysfunctions of sepsis, coagulopathy, capillary leak, and vasoplegia, with endothelial injury as a common latent factor.

Preliminary results indicate that easily obtainable clinical parameters, such as the SIC score, serum albumin levels, and peripheral vascular resistance, may provide valuable insights into the severity of underlying cellular alterations, which are currently not routinely assessable in clinical practice.

While this model is still preliminary and requires further validation, its ability to integrate clinically accessible parameters into a coherent pathophysiological framework represents an innovative approach that could support future efforts toward personalized sepsis care.

## Figures and Tables

**Table 1:** Clinical and demographic characteristics of the patients enrolled in the study.

Variables	
Patients, n (%)	75 (100)
Age, years, mean (SD)	69.2 (12.2)
Sex, n (%)	
Female	31 (41.3)
Male	44 (58.7)
Biometric data	
Height, cm, mean (SD)	171,3 (9.6)
Weight, kg, mean (SD)	78.4 (19.5)
BMI, kg/m^2^, mean (SD)	26.6 (6.4)
Vital signs	
Systolic BP, mmHg, mean (SD)	115.9 (25.5)
Diastolic BP, mmHg, media (SD)	71.5 (15.9)
Heart rate, bpm, median (IQR)	93 (79–105)
Respiratory rate, breaths/min, median (IQR)	20 (18–25)
Non-Invasive Hemodynamic Assessment (NICaS)	
Stroke Volume, mean (SD)	76.4 (20.1)
Stroke Volume Index, median (IQR)	40.2 (32.6–46.2)
Cardiac Output, median (IQR)	6.7 (5.8–8.5)
Cardiac Index, media (SD)	3.8 (1.2)
Cardiac Power Index, median (IQR)	0.69 (0.53–0.85)
TPR, median (IQR)	9.96 (7.61–1.17)
TPRI, mean (SD)	1.96 (0.73)
SOFA score, point, median (IQR)	4 (3–6)
Serum Albumin, g/dL, mean (SD)	2.6 (0.6)
SIC score, point, mean (SD)	3.5 (1.3)

**Table 2:** Structural equation modeling results assessing the association between endothelial damage markers and clinical parameters.

	β Coefficent	95% CI	Standard Error	*p*-Value	Residual Variance	95% CI
Serum Albumin	−0.416	(−0.240–−0.592)	0.090	<0.001	0.162	(0.076–0.348)
TPRI	−0.369	(−0.173–−0.565)	0.100	<0.001	0.401	(0.272–0.591)
SIC score	0.809	(0.444–1.174)	0.186	<0.001	0.923	(0.527–1.618)

**Table 3:** Correlation matrix showing the relationships between Serum Albumin, TPRI, and SIC-score.

	Serum Albumin	TPRI	SIC Score
Serum Albumin	-	0.361 *p* = 0.001	−0.462 *p* < 0.001
TPRI	0.361 *p* = 0.001	-	−0.324 *p* = 0.005
SIC score	−0.462 *p* < 0.001	−0.324 *p* = 0.005	-

**Table 4:** Comparison of clinical and hemodynamic variables across clusters identified through dendrogram-based hierarchical clustering. The table presents the distribution of patients, serum albumin levels, TPRI, SIC scores, and 30-day mortality rates across three distinct clusters.

Variables	Cluster 1	Cluster 2	Cluster 3	*p*-Value
Patients, n (%)	39 (52.0)	25 (33.3)	11 (14.7)	
Serum Albumin, g/dL, mean (SD)	2.4 (0.5)	2.6 (0.6)	3.0 (0.6)	0.004
TPRI, mean (SD)	1397 (318)	2273 (176)	3264 (466)	<0.001
SIC score, points, mean (SD)	3.7 (1.2)	3.0 (1.2)	3.1 (1.4)	0.018
30-day mortality, n (%)	7 (17.9)	0 (0.0)	1 (9.1)	0.059

## Data Availability

Data available on request, due to privacy/ethical restrictions.

## Supplementary Materials

The following supporting information can be downloaded at: https://www.mdpi.com/article/10.3390/clinpract15070120/s1, Supplementary File S1: Patients Enrollment Flowchart; Supplementary File S2: Statistical assumption of the SEM.
