# Supplementary material for: Endothelial Damage in Sepsis: The Interplay of Coagulopathy, Capillary Leak, and Vasoplegia—A Physiopathological Study
Source: Clin Pract. 2025 Jun 25;15(7):120. doi: 10.3390/clinpract15070120 (PMC12293470; doi:10.3390/clinpract15070120)
Supplement: Supplementary file 1 [file clinpract-15-00120-s001.zip › clinpract-3687068-supplementary.pdf]

clinics and practice

MDPI

Supplementary File S1: Patients Enrollment Flowchart

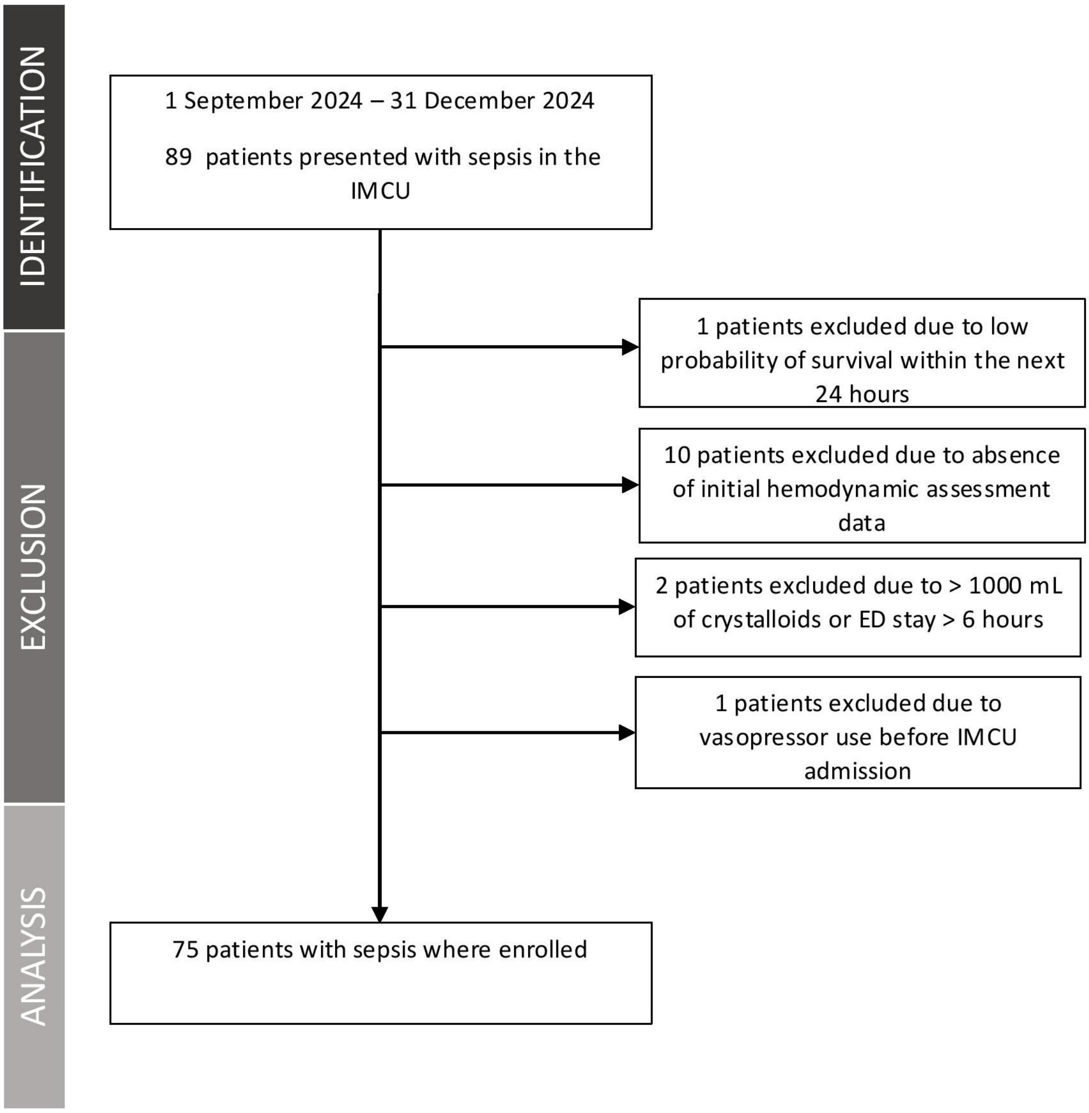

Supplementary File S2: Statistical assumption of the SEM

This supplementary file provides diagnostic assessments supporting the statistical assumptions of the SEM applied in the study. The focus is on verifying the distributional properties of the observed variables and on reporting the model's overall fit metrics.

Supplementary Table 1: Normality Assessment of Observed Variables

Variable	W Statistic	p-value
SIC	0.905	<0.001
Albumin	0.982	0.350
TPRI	0.962	0.237

Supplementary Table 2: Distribution Shape Descriptors: Skewness and Kurtosis

Variable	Skewness	Kurtosis
SIC	0.449	2.348
Albumin	0.030	3.262
TPRI	0.711	3.421

Supplementary Table 3: Fit indices for the structural equation model assessing the relationship between the latent factor (endothelial dysfunction) and the observed variables.

Fit Statistic	Value
**RMSEA**	0.000
**CFI**	1.000
**TLI**	1.000
**SRMR**	0.000
**CD (R^2)**	0.679
**AIC**	530.864
**BIC**	551.721
